# Description of *Cryptosporidium ornithophilus* n. sp. (Apicomplexa: Cryptosporidiidae) in farmed ostriches

**DOI:** 10.1186/s13071-020-04191-2

**Published:** 2020-07-08

**Authors:** Nikola Holubová, Lenka Tůmová, Bohumil Sak, Adéla Hejzlarová, Roman Konečný, John McEvoy, Martin Kváč

**Affiliations:** 1 Institute of Parasitology, Biology Centre of the Czech Academy of Sciences, v.v.i, České Budějovice, Czech Republic; 2 Faculty of Agriculture, University of South Bohemia in České Budějovice, České Budějovice, Czech Republic; 3 Veterinary and Microbiological Sciences Department, North Dakota State University, Fargo, USA

**Keywords:** *Cryptosporidium* avian genotype II, *Cryptosporidium ornithophilus* n. sp., *C. ubiquitum*, Occurrence, Oocyst size, PCR, Experimental infections

## Abstract

**Background:**

Avian cryptosporidiosis is a common parasitic disease that is caused by five species, which are well characterised at the molecular and biological level, and more than 18 genotypes for which we have limited information. In this study, we determined the occurrence and molecular characteristics of *Cryptosporidium* spp. in farmed ostriches in the Czech Republic.

**Methods:**

The occurrence and genetic identity of *Cryptosporidium* spp. were analysed by microscopy and PCR/sequencing of the small subunit rRNA, *actin*, *HSP70* and *gp60* genes. *Cryptosporidium* avian genotype II was examined from naturally and experimentally infected hosts and measured using differential interference contrast. The localisation of the life-cycle stages was studied by electron microscopy and histologically. Infectivity of *Cryptosporidium* avian genotype II for cockatiels (*Nymphicus hollandicus* (Kerr)), chickens (*Gallus gallus* f. *domestica* (L.)), geese (*Anser anser* f. *domestica* (L.)), SCID and BALB/c mice (*Mus musculus* L.) was verified.

**Results:**

A total of 204 individual faecal samples were examined for *Cryptosporidium* spp. using differential staining and PCR/sequencing. Phylogenetic analysis of small subunit rRNA, *actin*, *HSP70* and *gp60* gene sequences showed the presence of *Cryptosporidium* avian genotype II (*n* = 7) and *C. ubiquitum* Fayer, Santín & Macarisin, 2010 IXa (*n* = 5). Only ostriches infected with *Cryptosporidium* avian genotype II shed oocysts that were detectable by microscopy. Oocysts were purified from a pooled sample of four birds, characterised morphometrically and used in experimental infections to determine biological characteristics. Oocysts of *Cryptosporidium* avian genotype II measure on average 6.13 × 5.15 μm, and are indistinguishable by size from *C. baileyi* Current, Upton & Haynes, 1986 and *C. avium* Holubová, Sak, Horčičková, Hlásková, Květoňová, Menchaca, McEvoy & Kváč, 2016. *Cryptosporidium* avian genotype II was experimentally infectious for geese, chickens and cockatiels, with a prepatent period of four, seven and eight days post-infection, respectively. The infection intensity ranged from 1000 to 16,000 oocysts per gram. None of the naturally or experimentally infected birds developed clinical signs in the present study.

**Conclusions:**

The molecular and biological characteristics of *Cryptosporidium* avian genotype II, described here, support the establishment of a new species, *Cryptosporidium ornithophilus* n. sp.
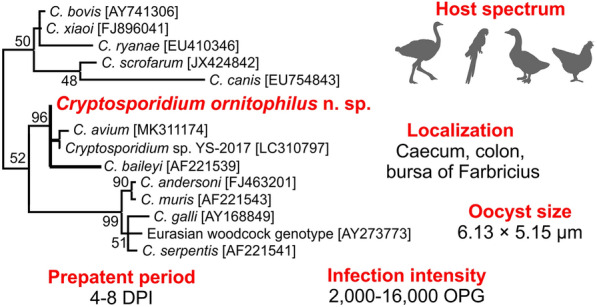

## Background

The genus *Cryptosporidium* Tyzzer, 1910 comprises protist parasites that infect epithelial cells in the microvillus border, primarily of the gastrointestinal tract, of all classes of vertebrates [1]. Until recently, only three bird-derived *Cryptosporidium* species, *C. baileyi* Current, Upton & Haynes, 1986, *C. galli* Pavlásek, 1999 and *C. meleagridis* Slavin, 1955, were described in birds [2–4]. Even with the recent descriptions of *C. avium* Holubová, Sak, Horčičková, Hlásková, Květoňová, Menchaca, McEvoy & Kváč, 2016 [5] and *C. proventriculi* Holubová, Zikmundová, Limpouchová, Sak, Konečný, Hlásková, Rajský, Kopacz, McEvoy & Kváč, 2019 [6], the number of described species in birds remains low relative to that in mammals. Eighteen *Cryptosporidium* genotypes (*Cryptosporidium* sp. YS-2017 genotype, avian genotype I, avian genotype IV, avian genotypes VI-IX, black duck genotype, Euro-Asian woodcock genotype, duck genotype, goose genotypes I-IV and goose genotype Id and finch genotypes I-III) have been identified [7–15], primarily based on small subunit rRNA sequence data, across 17 avian orders worldwide [8, 9, 13, 16, 17]. Although avian *Cryptosporidium* spp. have been studied more frequently in recent years, research has been biased towards *Cryptosporidium* in poultry and pet birds, with comparatively little attention paid to *Cryptosporidium* in other bird groups [16, 18].

Unlike *C. baileyi*, which infects a broad range of birds from different orders, many recently described *Cryptosporidium* species and genotypes appear to have a relatively narrow host range. For example, *Cryptosporidium* avian genotype VI appears to be restricted to North American red-winged blackbirds [8], and *Cryptosporidium* goose and duck genotypes have been found only in anseriform birds [11, 15]. Similarly, *C. avium* and *Cryptosporidium* avian genotype I are almost exclusively found in psittacines and passerines, respectively [5–7, 19]. *Cryptosporidium* avian genotype II has been found predominantly in ostriches but also in other species within the order Struthioniformes as well as orders Galliformes and Psittaciformes (Table 1).

**Table 1:** The occurrence of *Cryptosporidium* avian genotype II in birds from the orders Galliformes, Psittaciformes and Struthioniformes demonstrated on the basis of molecular tools amplifying partial sequences of *Cryptosporidium* small subunit ribosomal RNA (*SSU*), *actin* and 70 kDa heat-shock protein (*HSP70*) genes

Host	Country	Locus (GenBank ID)	No. positive/no. screened	Reference
Chicken (*Gallus gallus*)^a^	China	*SSU* (JX548291-92)	6/385	[57]
Ostrich (*Struthio camelus*)^c^	Vietnam	*SSU* (AB696811)	110/464	[36]
	Brazil	*SSU* (DQ002931) *Actin* (DQ002930) *HSP70* (DQ002929)	1/1 1/1 1/1	[59]
	Brazil	*SSU* (DQ650341)^d^ *Actin* (DQ650348)^d^	6/41 6/41	[19]
Cockatiel (*Nymphicus hollandicus*)^b^	Australia	*SSU* (DQ002931)^d^ *Actin* (DQ002930)^d^	3/ns 2/ns	[7]
Eclectus (*Eclectus roratus*)^b^	Australia	*SSU* (DQ650340) *Actin* (DQ650347)	2/ns 1/ns	[7]
Galah (*Eolophus roseicapilla*)	Australia	*SSU* (DQ650341) *Actin* (DQ650348)	1/ns 1/ns	[7]
Major Mitchell cockatoo (*Cacatua leadbeateri*)^b^	Australia	*SSU* (DQ002931)^d^ *Actin* (DQ002930)^d^	3/ns 1/ns	[7]
Alexandrine (*Psittacula eupatria*)^b^	Australia	*SSU* (DQ002931)^d^	1/ns	[7]
Princess parrot (*Polytelis alexandrae*)^b^	Australia	*SSU* (DQ002931)^d^	1/ns	[7]
Sun conure (*Aratinga solstitialis*)^b^	Australia	*SSU* (DQ002931)^d^	1/ns	[7]
White-eyed parakeet (*Aratinga leucophthalma*)^b^	Brazil	*SSU* (DQ650341)^d^	1/ns	[56]

^a^Galliformes

^b^Psittaciformes

^c^Struthioniformes

^d^The sequence obtained in the present study has not been stored in the GenBank database and was identical to sequence published previously

*Abbreviation*: ns, not specified

*Cryptosporidium* in ostriches was first reported in 1993 [20] and there have been several reports since then, although most have not described the molecular characteristics of isolates [20–31]. Where molecular studies have been performed, with the exception of the rodent-specific *C. muris* Tyzzer, 1907, which was detected in 22 birds [32], *C. baileyi* [4, 32–35] and *Cryptosporidium* avian genotype II [19, 36] have been the only *Cryptosporidium* spp. reported in ostriches. While the biology of *C. baileyi* is well studied, there is limited information about *Cryptosporidium* avian genotype II.

In the present study, we report on the occurrence of *Cryptosporidium* spp. in farmed ostriches. For the most prevalent genotype in ostriches, *Cryptosporidium* avian genotype II, we further describe oocyst morphometry, experimental host specificity, developmental stage localization and molecular characteristics. Based on the collective data from this and previous studies, we conclude that *Cryptosporidium* avian genotype II is genetically and biologically distinct from the species of *Cryptosporidium* considered valid, and propose the name *Cryptosporidium ornithophilus* n. sp. for this genotype.

## Methods

### Specimens studied

Faecal samples were collected from ostriches on four farms in the Czech Republic. Faecal samples from juvenile (aged 9–12 months) and adult (older than three years) ostriches were individually collected into sterile plastic vials and stored at 4–8 °C until subsequent processing. Faecal smears were prepared from each sample, stained with aniline-carbol-methyl violet (ACMV), and examined for the presence of *Cryptosporidium* spp. oocysts [37]. Faecal samples were also screened for the presence of *Cryptosporidium-*specific DNA by PCR/sequencing (described below). Oocysts of *C. ornithophilus* n. sp. were purified from pooled faecal samples from a naturally infected juvenile common ostrich (no. 43588, *Struthio camelus* L.) kept on the farm number 4 using caesium chloride gradient centrifugation [38]. Purified oocysts were used for morphometry and preparation of the inoculum. The propidium iodide (PI) staining was used for test of oocysts viability [39]. *Cryptosporidium ornithophilus* n. sp. oocysts from a common ostrich were pooled and used to infect a single one-day-old chickens (chicken 0; *Gallus gallus* f. *domestica*). Oocysts recovered from the faeces of chicken 0 were used to infect other experimental animals. The purity of *C. ornithophilus* n. sp. isolate before performing the experimental infection and taking the measurements, and during the experiments was verified by the following procedure. The sequence of the original isolate (ostrich) was compared to the sequence obtained from chicken 0 and from tissue specimens and faecal samples of experimentally inoculated animals (below). The oocyst size of the original isolate was compared with isolates obtained from susceptible hosts.

### Oocyst morphometry

Oocysts of *C. ornithophilus* n. sp. from naturally and experimentally infected hosts (50 oocysts from each isolate) were examined and length and width measurements were taken using differential interference contrast (DIC) at 1000× magnification. All measurements are in micrometres and are given as the range followed by the mean ± standard deviation (SD) in parentheses. These measurements were used to calculate the length-to-width ratio. Sample containing purified *C. parvum* Tyzzer, 1912 oocysts from a naturally infected Holstein calf was used as a size control (*n* = 50). Size of oocysts was measured using the same microscope and by the same person. Each slide was screened a meandering path to prevent repeated measurement of an oocyst. Additionally, different staining methods were used for visualisation of oocysts. Faecal smears with *C. ornithophilus* n. sp. and *C. parvum* (data not shown) oocysts were stained by ACMV, modified Ziehl-Neelsen [ZN; 40], phenol staining [AP; 41] and labelled with genus-specific FITC-conjugated antibodies (IFA; *Cryptosporidium* IF Test, Crypto cel, Cellabs Pty Ltd., Brookvale, Australia). Morphometry was determined using digital analysis of images (Olympus cellSens Entry 2.1 software and Olympus Digital Colour camera DP73, Olympus Corporation, Shinjuku, Tokyo, Japan). Photomicrographs of *C. ornithophilus* n. sp. oocysts observed by DIC, ACMV, ZN, AP and IFA were stored at the Institute of Parasitology, Biology Centre of the Czech Academy of Sciences, Czech Republic.

### Molecular analyses

Total genomic DNA was extracted from 20,000 purified oocysts, 200 mg of faeces, or 200 mg of tissue by bead disruption for 60 s at 5.5 m/s using 0.5 mm glass beads in a FastPrep®24 Instrument (MP Biomedicals, CA, USA) followed by isolation/purification using Exgene^TM^ Stool DNA mini (GeneAll Biotechnology Co. Ltd, Seoul, Korea) or DNeasy Blood & Tissue Kit (Qiagen, Hilden, Germany) in accordance with the manufacturer’s instructions. Purified DNA was stored at − 20 °C. A nested PCR approach was used to amplify a partial region of the small subunit (*SSU*) rRNA [42, 43], *actin* [44], 70 kilodalton heat-shock protein (*HSP70*) [45] and *gp60* [46–48] genes. The PCR conditions were slightly modified, for more details see [6]. Molecular grade water and DNA of *C. parvum* were used as negative and positive controls, respectively. Secondary PCR products were detected in 1.5% agarose gel stained with ethidium bromide. PCR products were cut out from gel, purified using Gen Elute Gel Extraction Kit (Sigma, St. Louis, MO, USA) and sequenced in both directions with an ABI 3130 genetic analyser (Applied Biosystems, Foster City, CA) using the secondary PCR primers in commercial laboratory (SEQme, Dobříš, Czech Republic).

### Phylogenetic analyses

The nucleotide sequences obtained in this study were edited using the ChromasPro 2.4.1 software (Technelysium, Pty, Ltd., South Brisbane, Australia) and aligned with reference sequences downloaded from GenBank using MAFFT version 7 online server (http://mafft.cbrc.jp/alignment/software/). The most appropriate evolutionary models for phylogeny analyses and values of all parameters for each model were selected using the MEGAX software [49, 50]. The evolutionary history was inferred by using the Maximum Likelihood (ML) method based on the Tamura 3-parameter model [51] selected for *SSU* and *HSP70* alignments and the general time reversible model [52] was selected for actin alignment. The trees with the highest log likelihood were shown. Bootstrap support for branching was based on 1000 replications. Phylogenetic trees obtained from the MEGAX (https://www.megasoftware.net/) were edited in CorelDrawX7 (https://www.coreldraw.com). Sequences of *SSU* (MN969954-MN969968), *actin* (MN973944-MN973958), *HSP70* (MN973934-MN973943) and *gp60* (MN973959-MN973963) generated in this study were deposited in the GenBank database.

### Animals for transmission studies

Five adult cockatiels (*Nymphicus hollandicus* (Kerr)), five one-day-old chickens, five one-day-old geese (*Anser anser* f. *domestica* L.), five seven-day- and eight-week-old SCID mice (*Mus musculus*; strain C.B-17) and five seven-day and eight-week-old BALB/c mice were used for transmission studies. Three adult cockatiels, chickens, geese and seven-day and eight-week-old SCID and BALB/c mice used as a negative control. As a control, the infectivity of *C. parvum* from a naturally infected Holstein calf for three adult cockatiels, chickens, geese and seven-day and eight-week-old SCID and BALB/c mice was verified. All animals, except chickens, geese and seven-day-old mice, which were hatched under laboratory conditions, were screened every other day for the presence of oocysts of *Cryptosporidium* spp. and specific DNA two weeks prior to transmission studies. Cockatiels originated from breeders located in the Czech Republic and laboratory mice were obtained from Charles River (Germany).

### Animal care

Rodents were individually housed in ventilated cages (Tecniplast, Buguggiate, Italy). Chickens and geese were housed in boxes and cockatiels were kept in separate aviaries. The size of boxes and aviaries were according to regulated by Czech legislation (Act No 246/1992 Coll., on protection of animals against cruelty). An external source of heat was used in the first five days for chickens and geese. Sterilized diet and water were available for all animals *ad libitum*. Animal caretakers wore sterile shoe covers and disposable coveralls and disposable gloves always they entered the experimental room. Wood-chip bedding and disposable protective clothing were removed from the experimental room and incinerated.

### Experimental design

A total 20,000 purified oocysts of *C. ornithophilus* n. sp., suspended in 10 µl of distilled water, were dropped into the mouth/beak of each animal. Animals serving as negative controls were inoculated orally with 10 µl of distilled water. Faecal samples from all animals were screened daily for the presence of *Cryptosporidium* oocysts using ACMV staining and the presence of *Cryptosporidium*-specific DNA was confirmed using nested PCR/sequencing targeting the *SSU* gene. All experiments were terminated 30 days post-infection (dpi). Infection intensity was reported as the number of oocysts per gram (opg) of faeces, as previously described by Kváč et al. [53]. In addition, faecal consistency and colour and general health status were examined daily. The sequence identity of the *Cryptosporidium* DNA recovered from infected hosts to inoculum and original isolate at *SSU*, *actin* and *HSP70* was verified in each experimentally infected animal.

### Histopathological and scanning electron microscopy (SEM) examinations

Two animals from each group (at 10 and 20 dpi) were examined at necropsy. Tissue samples from oesophagus; stomach in rodents and proventriculus and ventriculus in birds; duodenum; jejunum (proximal, central and distal); ileum; caecum and colon were collected for histology and SEM followed by processing according [6]. Slides for histology were examined at 100–400× magnification and documented using Olympus cell Sens Entry 2.1 (Olympus Corporation, Shinjuku, Tokyo, Japan) equipped with a digital camera (Olympus DP73). Samples for SEM were examined using a JEOL JSM-7401F-FE SEM and documented using ETD Detector A PRED (Thermo Fisher Scientific, Waltham, MA, USA). Additionally, DNA from tissue samples was isolated and the sequence identity to inoculum and original isolate at *SSU*, *actin* and *HSP70* was verified.

### Staining of mucosal smears

Wright staining procedures were used to visualize *Cryptosporidium* spp. developmental stages in the gastrointestinal tract of chickens [54]. Tissue samples of the large intestine (selected on the basis of histological examination) were washed with cold PBS with subsequent exposure to serum from *Cryptosporidium-*negative chickens for five min. The mucous membrane was gently scrapped with a scalpel and smeared on a glass slide. Wet mucosal smears were fixed with osmium vapour for 15 min followed by Wright staining for 6 min. Slides were viewed at 1000× magnification and documented using Olympus cell Sens Entry 2.1 (Olympus Corporation, Shinjuku, Tokyo, Japan) equipped with a digital camera (Olympus DP73).

### Statistical analysis

Differences in *Cryptosporidium* spp. oocysts size were tested using Hotellingʼs multivariate version of the 2 sample t-test, *package ICSNP: Tools for Multivariate Nonparametrics* in R 4.0.0. [55]. The hypothesis tested was that two-dimensional mean vectors of measurement are the same in the two populations being compared.

## Results

A total of 164 juvenile and 40 adult ostriches were screened for the presence of *Cryptosporidium* infection. *Cryptosporidium* spp. was detected on three out of four ostrich farms. Out of 204 faecal samples, five (2.5%) were microscopically positive for the presence of *Cryptosporidium* oocysts and 12 (5.9%) contained specific DNA of *Cryptosporidium* spp. (Table 2). All microscopically positive samples were also positive for *Cryptosporidium* DNA. Only juvenile ostriches (*n* = 12) were infected with *Cryptosporidium* spp. Screened animals had good health and faecal consistency appropriate to the age of birds and feeding.

**Table 2:** *Cryptosporidium* species and genotypes from this study, detected by amplification of small subunit ribosomal rRNA (*SSU*), *actin*, 70 kDa heat-shock protein (*HSP70*) and 60 kDa glycoprotein (*gp60*) gene fragments in juvenile common ostriches (*Struthio camelus*) on commercial farms in the Czech Republic

Farm No.	No. of positive/no. of screened	ID of positive animal	Microscopical positivity (opg)	Genotyping at the gene loci			
				*SSU*	*Actin*	*HSP70*	*gp60*
1	5/40	43201	No	*C. ubiquitum*	*C. ubiquitum*	–	XIIa
		43205	No	*C. ubiquitum*	*C. ubiquitum*	–	XIIa
		43210	No	*C. ubiquitum*	*C. ubiquitum*	–	XIIa
		43223	No	*C. ubiquitum*	*C. ubiquitum*	–	XIIa
		43228	No	*C. ubiquitum*	*C. ubiquitum*	–	XIIa
2	0/64	–	–	–	–	–	–
3	3/50	44782	Yes (8000)	*C. ornitophilus* n. sp.	*C. ornitophilus* n. sp.	*C. ornitophilus* n. sp.	–
		44790	No	*C. ornitophilus* n. sp.	*C. ornitophilus* n. sp.	*C. ornitophilus* n. sp.	–
		44796	Yes (12,000)	*C. ornitophilus* n. sp.	*C. ornitophilus* n. sp.	*C. ornitophilus* n. sp.	–
4	4/50	43545	Yes (12,000)	*C. ornitophilus* n. sp.	*C. ornitophilus* n. sp.	*C. ornitophilus* n. sp.	–
		43551	No	*C. ornitophilus* n. sp.	*C. ornitophilus* n. sp.	*C. ornitophilus* n. sp.	–
		43587	Yes (6000)	*C. ornitophilus* n. sp.	*C. ornitophilus* n. sp.	*C. ornitophilus* n. sp.	–
		43588	Yes (18,000)^a^	*C. ornitophilus* n. sp.	*C. ornitophilus* n. sp.	*C. ornitophilus* n. sp.	–

^a^Animal serving as a source of oocysts for transmission studies

*Note*: Infection intensity of *Cryptosporidium* spp. is expressed as the number of oocysts per gram of faeces (opg)

**Fig. 1:**
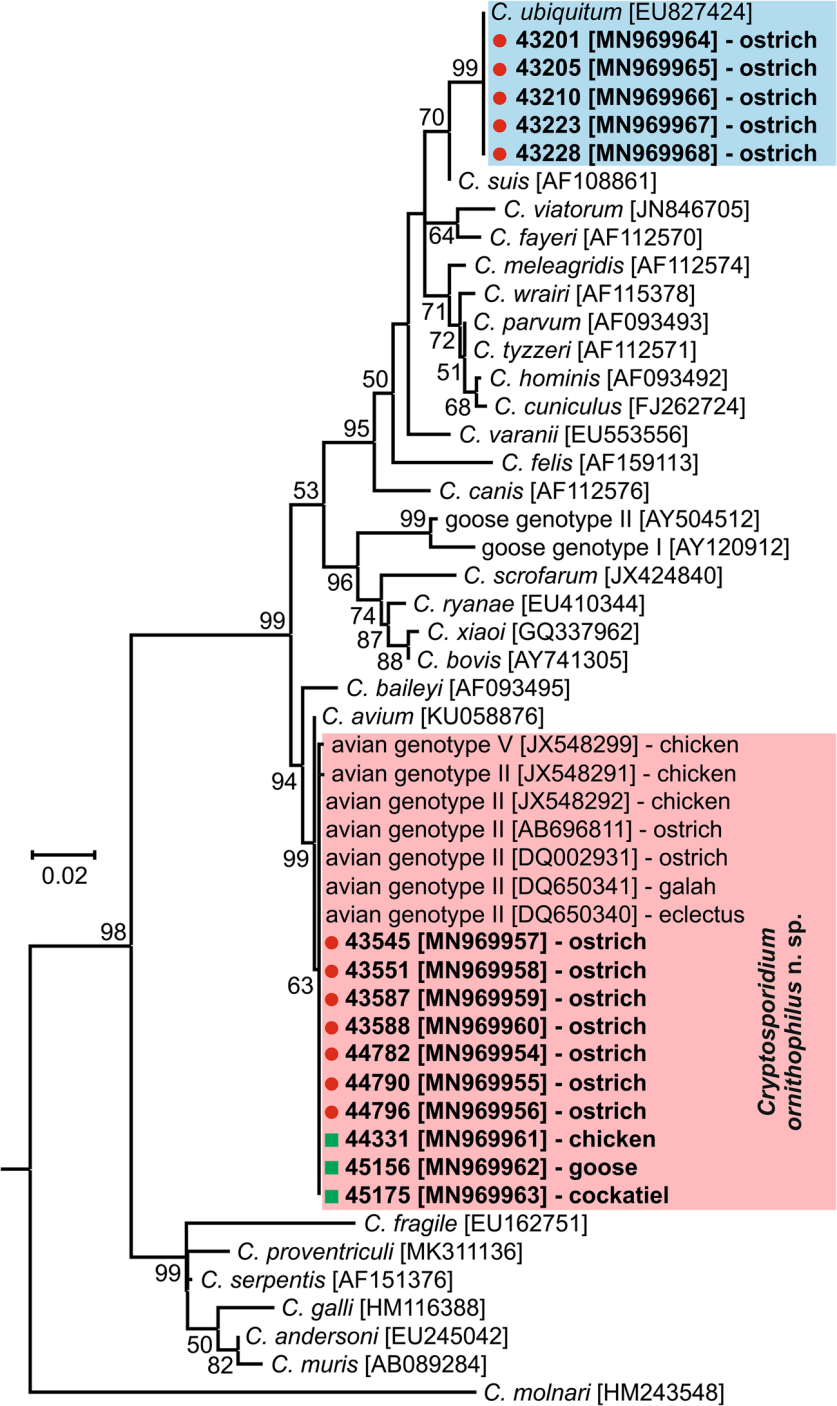
Maximum likelihood tree (− ln = 3130.05) based on partial sequences of the gene encoding the small subunit rRNA (*SSU*), including sequences obtained in this study from naturally (red circle and bolded) and experimentally (green square and bolded) infected hosts. Tamura’s 3-parameter model was applied, using a discrete Gamma distribution and invariant sites. The robustness of the phylogeny was tested with 1000 bootstrap pseudoreplicates and numbers at the nodes represent the bootstrap values > 50%. The scale-bar indicates the number of substitutions per site. Sequences obtained in this study are identified by isolate number (e.g. 43201)

**Fig. 2:**
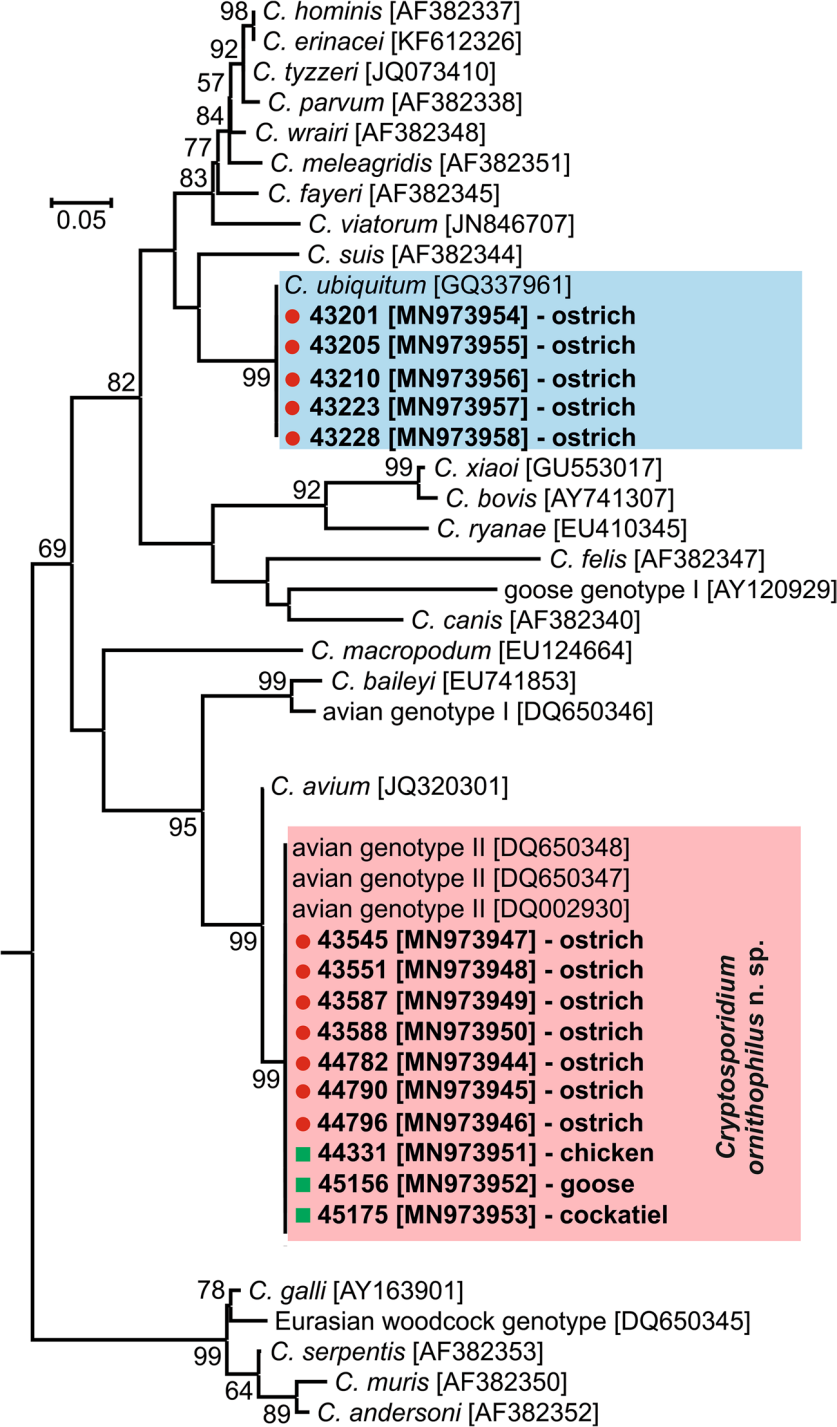
Maximum likelihood tree (− ln = 3641.49) based on partial sequences of the actin gene, including sequences obtained in this study from naturally (red circle and bolded) and experimentally (green square and bolded) infected hosts. The General Time Reversible model was applied, using a discrete Gamma distribution and invariant sites. The robustness of the phylogeny was tested with 1000 bootstrap pseudoreplicates and numbers at the nodes represent the bootstrap values > 50%. The scale-bar indicates the number of substitutions per site. Sequences obtained in this study are identified by isolate number (e.g. 43201)

**Fig. 3:**
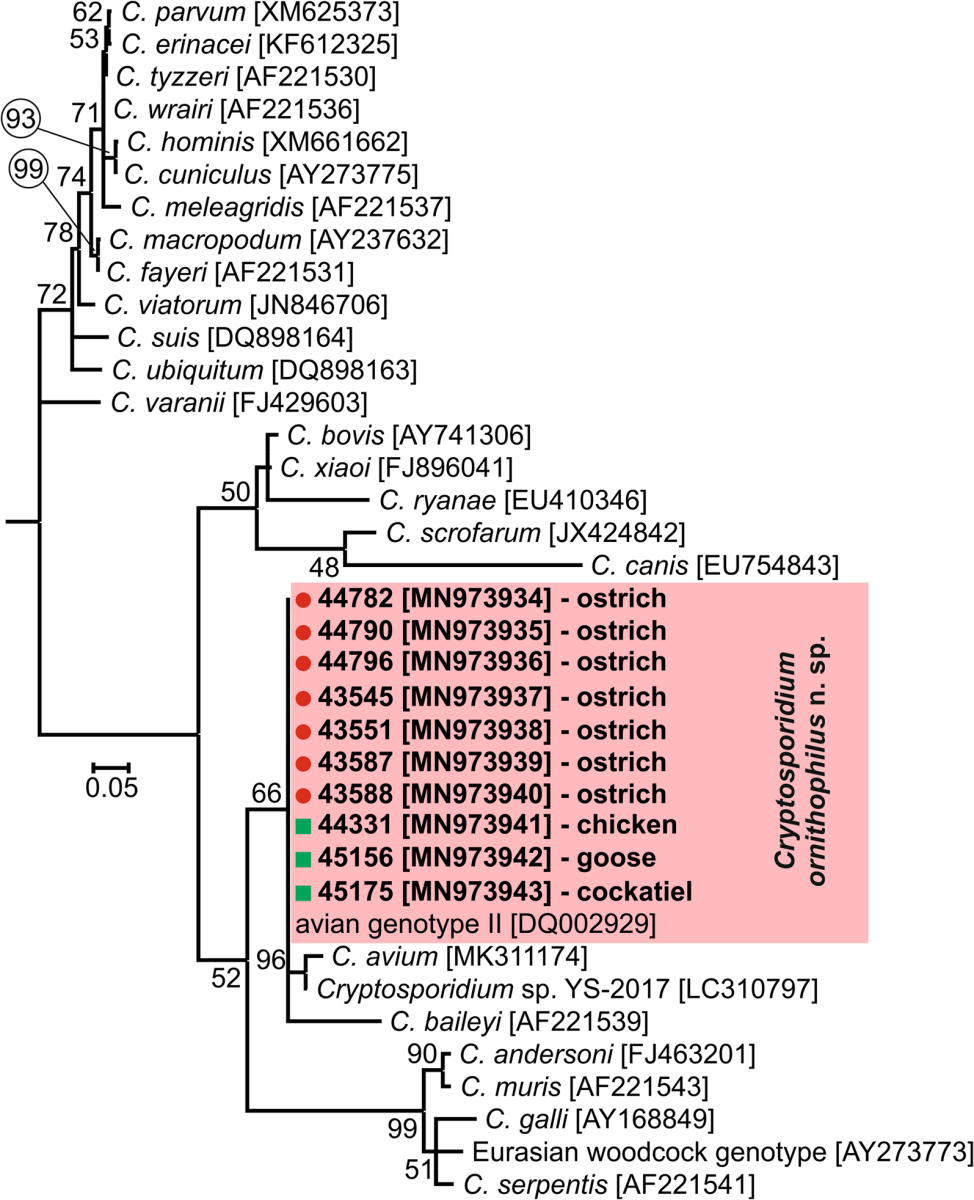
Maximum likelihood tree (− ln = 2009.56) based on partial sequences of the 70 kDa heat-shock protein gene, including sequences obtained in this study from naturally (red circle and bolded) and experimentally (green square and bolded) infected hosts. Tamura’s 3-parameter model was applied, using a discrete Gamma distribution and invariant sites. The robustness of the phylogeny was tested with 1000 bootstrap pseudoreplicates and numbers at the nodes represent the bootstrap values > 50%. The scale-bar indicates the number of substitutions per site. Sequences obtained in this study are identified by isolate number (e.g. 43201)

**Fig. 4:**
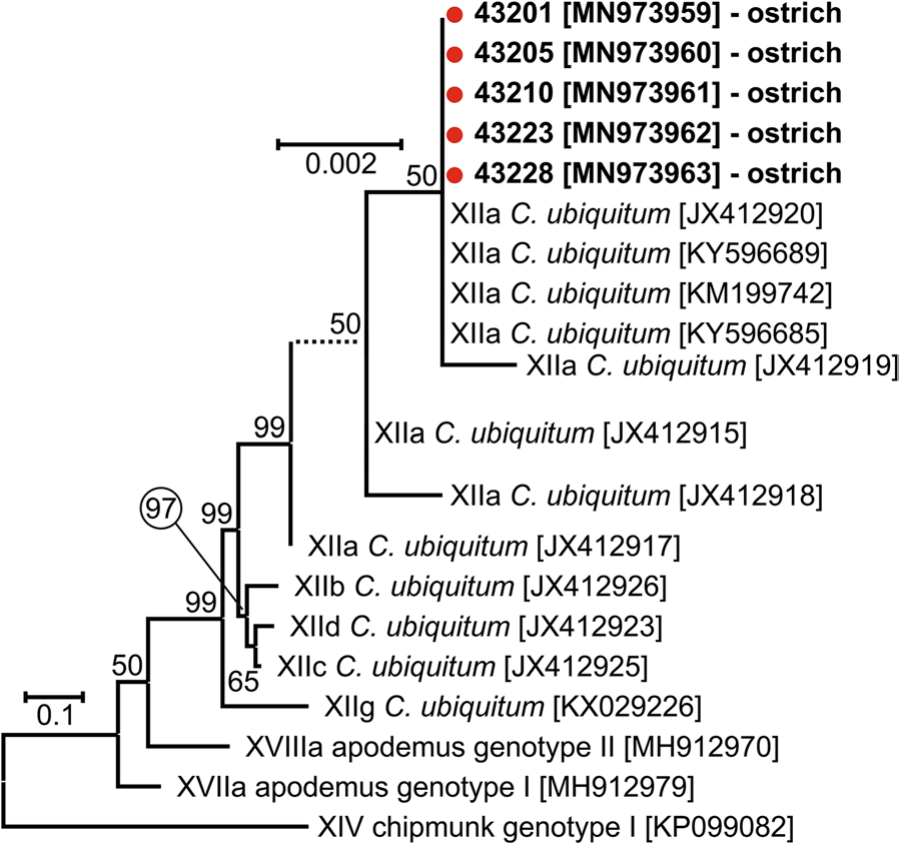
Maximum likelihood tree (− ln = 4017.25) based on partial sequences of the 60 kDa glycoprotein gene of *Cryptosporidium ubiquitum, Cryptosporidium* apodemus genotype I and II and *Cryptosporidium* chipmunk genotype I, including sequences obtained in this study from naturally infected hosts (red circles and bolded). Tamura’s 3-parameter model was applied, using invariant sites. The robustness of the phylogeny was tested with 1000 bootstrap pseudoreplicates and numbers at the nodes represent the bootstrap values > 50%. The scale-bar indicates the number of substitutions per site. Sequences obtained in this study are identified by isolate number (e.g. 43201)

All birds positive for *Cryptosporidium-*specific DNA were successfully genotyped by sequence analysis of *SSU* and *actin* genes (Table 2). ML trees constructed from *SSU* and *actin* sequences in this study showed the presence of *C. ubiquitum* Fayer, Santín & Macarisin, 2010 (*n* = 5) and *C. ornithophilus* n. sp. (*n* = 7; Table 2, Figs. 1, 2). *HSP70* gene sequences were successfully amplified only from samples positive for *C. ornithophilus* n. sp. (Fig. 3). The *C. ubiquitum gp60* gene was amplified and sequenced from five positive DNA samples from farm no. 1 (Table 2, Fig. 4). Sequences were identical to each other and clustered with subtype family XIIa (Fig. 4). Out of seven ostriches positive for *C. ornithophilus* n. sp., five shed microscopically detectable oocysts (6000–18,000 opg, Table 2). Birds positive for *C. ubiquitum* DNA did not shed oocysts detectable by microscopy.

**Fig. 5:**
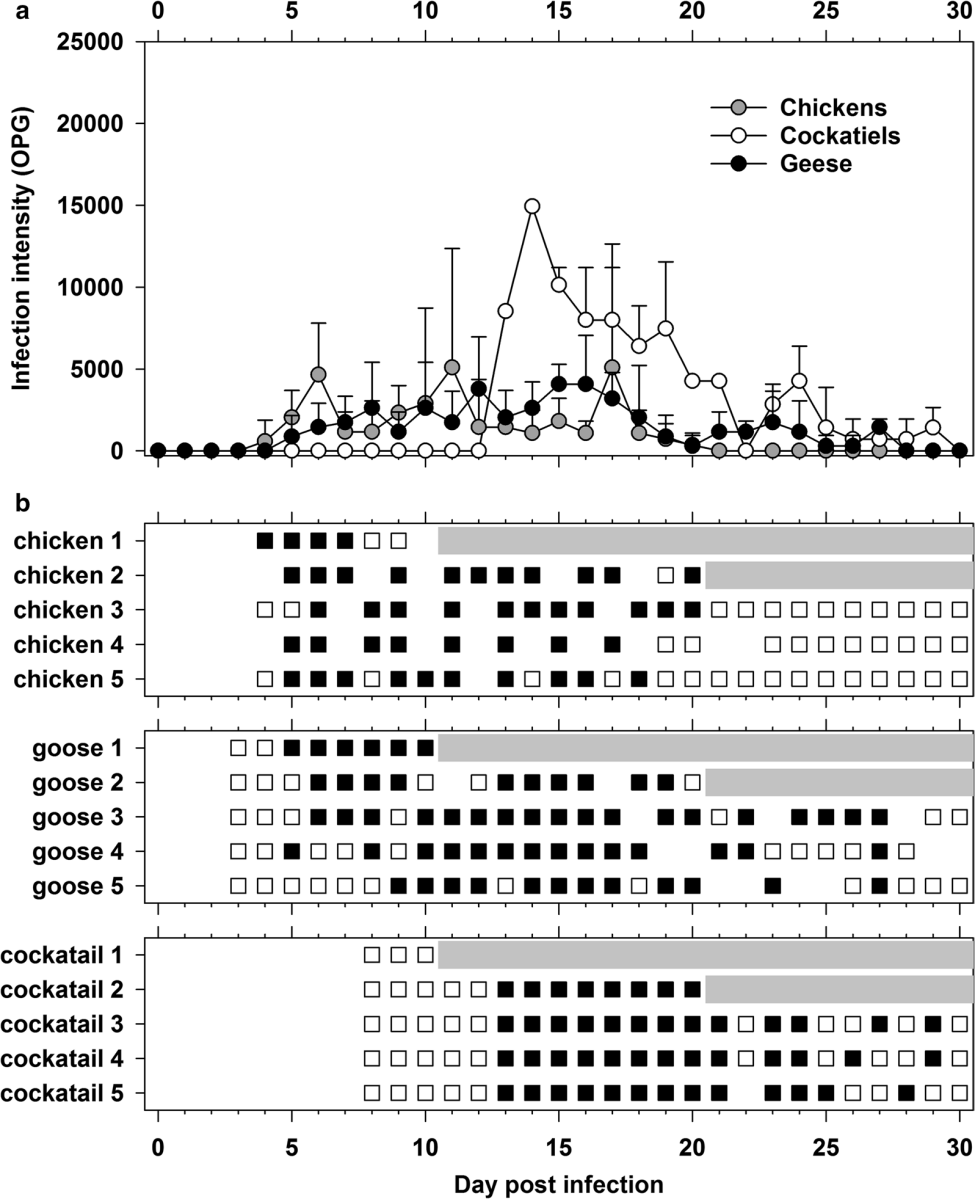
Course of infection of *Cryptosporidium ornitophilus* n. sp. in experimentally infected chickens (*Gallus gallus* f. *domestica*), geese (*Anas platyrhynchos* f. *domestica*) and cockatiels (*Nymphicus hollandicus*). **a** Infection intensity as number of oocysts per gram of faeces (opg). **b** Daily shedding of *C. ornitophilus* n. sp. based on coprological and molecular examination of faeces. Open squares indicate detection of specific DNA; filled squares indicate detection of oocysts by microscopy; grey rectangles indicate sacrifice and dissection of animal

*Cryptosporidium ornithophilus* n. sp. oocysts did not infect 7-day-old and 8-week-old BALB/c or SCID mice, whereas 7-day-old BALB/c and both age categories of SCID mice were infected with *C. parvum* (control group, data not shown). All chickens, geese and cockatiels inoculated with oocysts of *C. ornithophilus* n. sp. developed infections. Oocysts or specific DNA were first detected at 4 dpi, 7 dpi and 8 dpi in geese, chickens and cockatiels, respectively (Fig. 5). The infection intensity ranged from 2000 to 16,000 opg in chickens and cockatiels and from 1000 to 8000 opg in geese (Fig. 5).

**Fig. 6:**
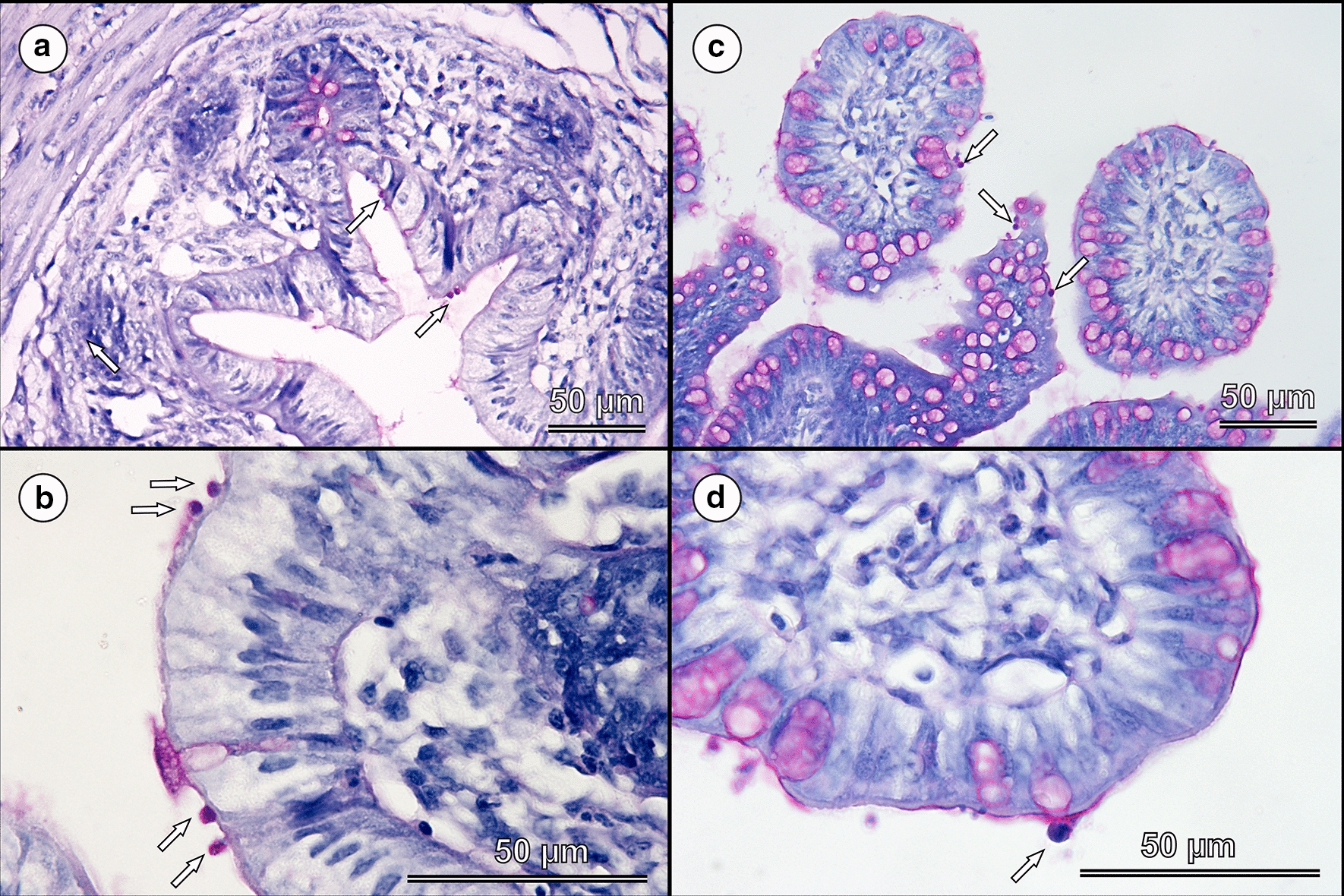
Histology sections of the caecum (**a** and **b**) and colon (**c** and **d**) of a chicken (*Gallus gallus* f. *domestica*) experimentally infected with 20,000 oocysts of *Cryptosporidium ornitophilus* n. sp., sacrificed 10 days post-infection. Attached developmental stages of *C. ornitophilus* n. sp. are indicated by arrows. Periodic Acid-Schiff (PAS) staining. *Scale-bars*: 50 µm

**Fig. 7:**
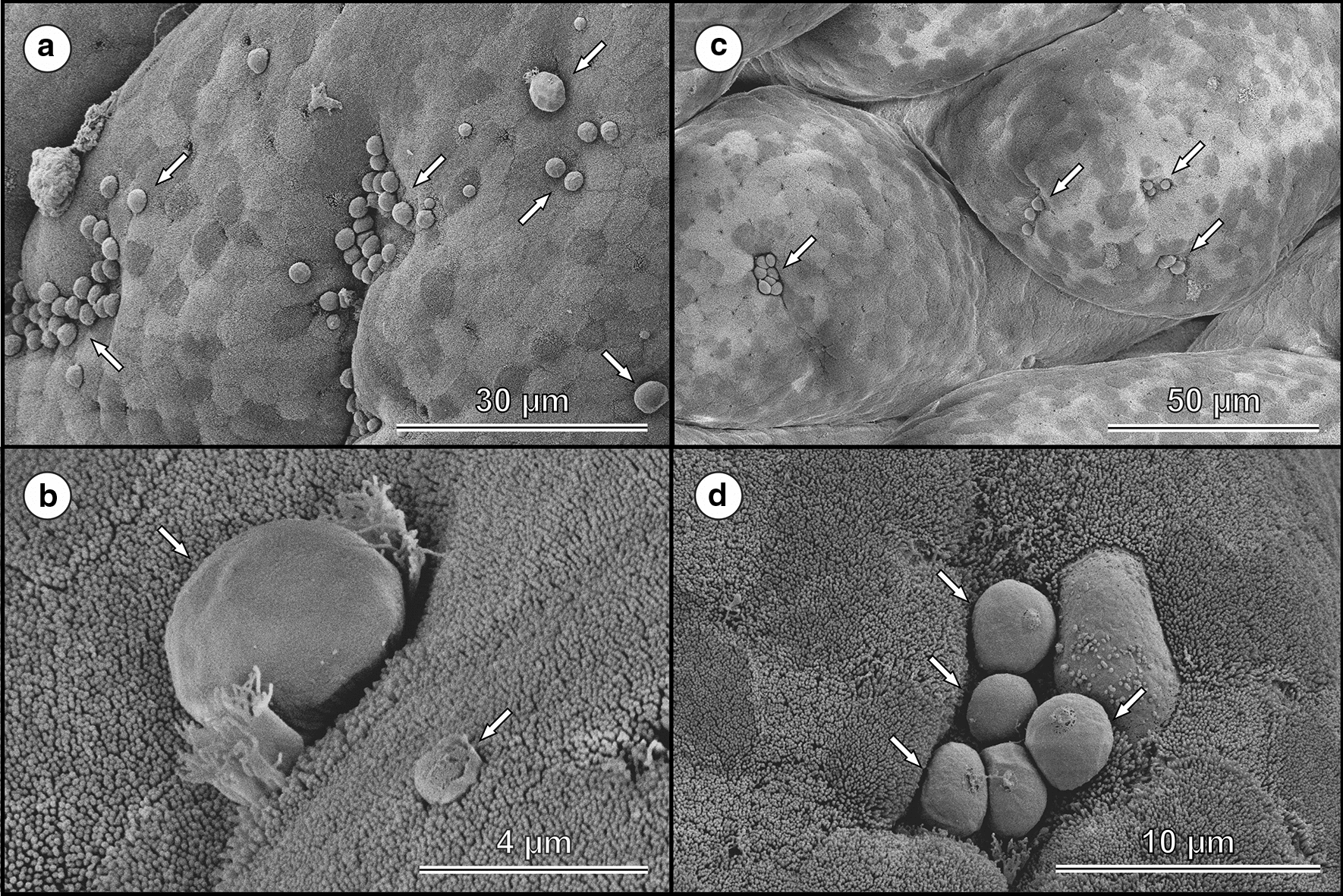
Scanning electron micrographs of developmental stages of *Cryptosporidium ornitophilus* n. sp. (arrows) on the epithelia surface of the caecum (**a** and **b**) and colon (**c** and **d**) of a chicken (*Gallus gallus* f. *domestica*) experimentally infected with 20,000 oocysts and sacrificed 10 days post-infection

Molecular, histological and SEM analyses and examination of stained mucosal smears of gastrointestinal tract tissue in birds with *C. ornithophilus* n. sp. showed the presence of developmental stages only in the caecum and colon of chickens and geese sacrificed 10 and 20 dpi (Figs. 6, 7). Few developmental stages were detected on each villus (Figs. 6, 7). Developmental stages were not detected in cockatiels, but specific DNA was detected exclusively in the caudal part of the ileum.

**Fig. 8:**
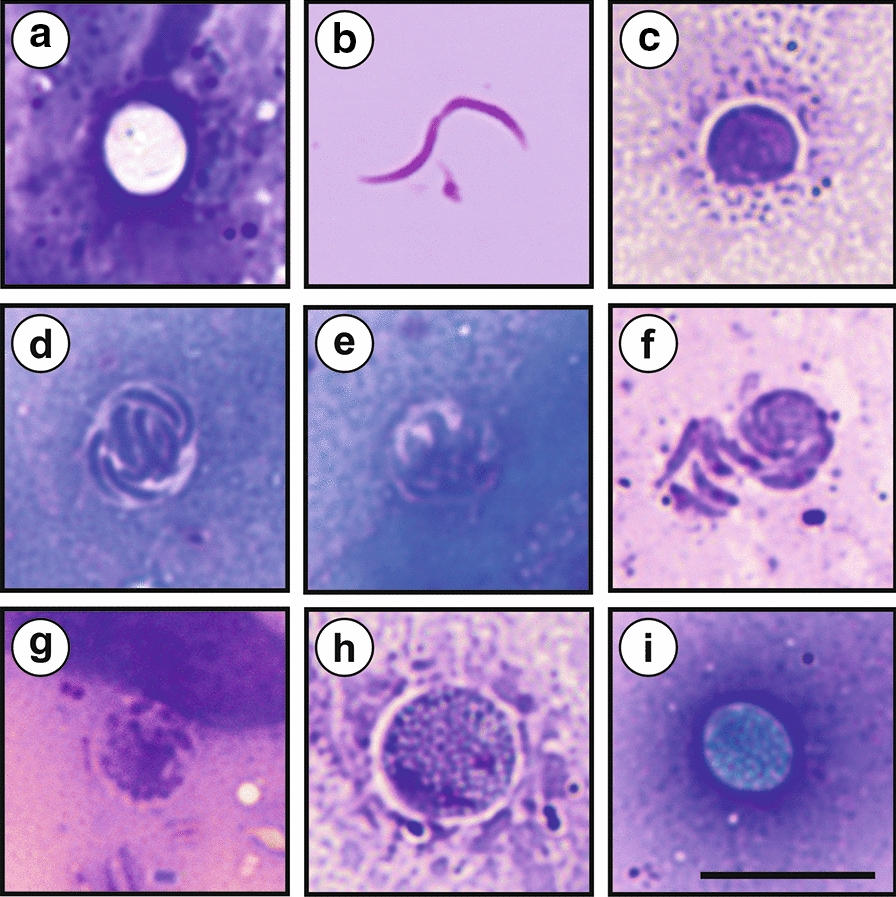
Developmental stages of *Cryptosporidium ornitophilus* n. sp. obtained from the colon of chickens (*Gallus gallus* f. *domestica*) experimentally infected with 20,000 oocysts and sacrificed 10 days post-infection. **a** Oocyst. **b** Sporozoite. **c** Mononuclear trophozoite. **d** Type I meront. **e** Type II meront. **f** Merozoites. **g** Microgamont. **h** Macrogamont. **i** Zygote. *Scale-bar*: 10 μm

The morphometry of the developmental stages of *C. ornithophilus* n. sp. was examined in preparations with Wright’s stain (Table 3). Most of the detected developmental stages were enveloped by a parasitophorous sac, which appeared as an unstained halo (Fig. 8). A large number of oocysts was detected, and most were unstained with sporozoites not visible (Fig. 8). We were not able to differentiate between thin- and thick-walled oocysts. Free sporozoites were not detected, but a photomicrograph of sporozoites following oocyst excystation is included in Fig. 8. Mononuclear trophozoites were the most frequently observed developmental stage which also showed a high variability in size (Fig. 8; Table 3). Type I meronts, containing 8 merozoites, were observed frequently (Fig. 8), while Type II meronts, with 4 merozoites, were found rarely (Fig. 8). Free merozoites were found rarely (Fig. 8). Microgamonts were found rarely (Fig. 8), but macrogamonts, typified by a number of amylopectin granules in their cytoplasm and a foam-like appearance, were frequently observed (Fig. 8). Zygotes were lightly stained compared to the unstained oocysts (Fig. 8).

**Table 3:** Size of developmental stages of *Cryptosporidium ornitophilus* n. sp. obtained from the colon of an experimentally infected chicken (*Gallus gallus* f. *domestica*) with 200,000 oocysts and sacrificed 20 days post-infection

Developmental stage	Length (µm)	Width (µm)
	Range (Mean ± SD)	Range (Mean ± SD)
Oocyst	5.24–6.74 (6.13 ± 0.34)	4.71–5.48 (5.21 ± 0.23)
Sporozoite	5.47–6.57 (6.07 ± 0.32)	0.54–0.63 (0.59 ± 0.02)
Trophozoite	2.56–6.40 (4.36 ± 1.16)	2.16–5.50 (3.90 ± 1.05)
Early Type I meront	4.95–6.54 (5.96 ± 0.57)	4.00–5.79 (5.12 ± 0.64)
Late Type I meront	6.66–8.94 (7.50 ± 0.84)	5.72–7.11 (6.40 ± 0.44)
Type II meront	6.60–6.78 (6.67 ± 0.10)	6.37–6.54 (6.48 ± 0.10)
Merozoite	4.61–5.41 (5.05 ± 0.11)	0.62–0.95 (0.77 ± 0.33)
Macrogamont	5.29–8.87 (6.70 ± 0.97)	4.32–8.13 (6.10 ± 1.09)
Microgamont	6.14–6.92 (6.54 ± 0.23)	6.02–6.73 (6.39 ± 0.23)
Zygote	5.56–6.83 (6.14 ± 0.45)	4.19–5.79 (5.20 ± 0.62)

*SSU*, *actin* and *HSP70* sequences obtained from the original isolate of *C. ornithophilus* n. sp. (ostrich) were identical to isolates recovered from faeces of chicken 0 and all other birds infected during the whole experiment. Additionally, sequences obtained from the tissue specimens of caecum and colon of chickens and geese and in the ileum of cockatiels were also identical to the inoculum. The gene encoding *gp60* was not successfully amplified in any animal experimentally infected with *C. ornithophilus* n. sp., indicating the absence of *C. ubiquitum* or other species and genotypes of *Cryptosporidium* spp. (e.g. *C. parvum*) that could be part of the inoculum.

The above data tend to justify the distinct status of *Cryptosporidium ornitophilus* n. sp., which is described below.

**Family Cryptosporidiidae Léger, 1911**

**Genus*Cryptosporidium*Tyzzer, 1907**

***Cryptosporidium ornitophilus*n. sp.**

Syn. *Cryptosporidium* sp. ex *Struthio camelus* 2005 of Meireles et al. [59]; *Cryptosporidium* avian genotype II of Ng et al. [7], Nguyen et al. [36] and Sevá et al. [56]

***Type-host***: *Struthio camelus* Linnaeus (Struthioniformes: Struthionidae), common ostrich.

***Other natural hosts***: Alexandrine (*Psittacula eupatria* (L.)) (as *Cryptosporidium* avian genotype II [7]), chicken (*Gallus gallus* f*. domestica*) (as *Cryptosporidium* avian genotype II [57]), cockatiel (*Nymphicus hollandicus*) (as *Cryptosporidium* avian genotype II [7]), eclectus (*Eclectus roratus* (Müller)) (as *Cryptosporidium* avian genotype II [7]), galah (*Eolophus roseicapilla* (Vieillot)) (as *Cryptosporidium* avian genotype II [7]), Major Mitchell cockatoo (*Cacatua leadbeateri* (Vigors)) (as *Cryptosporidium* avian genotype II [7]), princess parrots (*Polytelis alexandrae* (Gould)) (as *Cryptosporidium* avian genotype II [7]), sun conure (*Aratinga solstitialis* (L.)) (as *Cryptosporidium* avian genotype II [7]), white-eyed parakeet (*Aratinga leucophthalma* (Statius Müller)) (as *Cryptosporidium* avian genotype II [56]).

***Experimentally susceptible host***: *Gallus gallus* f. *domestica* L. (Galliformes: Phasianidae), chicken; *Anser anser* f. *domestica* L. (Anseriformes: Anatidae), goose; *Nymphicus hollandicus* (Kerr) (Psittaciformes: Cacatuidae), cockatiel.

***Type-locality***: Ostrich farm at Židovice (50.4451578N, 14.2297606E), Czech Republic.

***Other locality***: Ostrich farm at Fulnek (49.7123761N, 17.9031931E) Czech Republic.

***Type-material***: Tissue samples in 10% formaldehyde and histological sections of infected cecum (no. 2/2019) and colon (no. 3/2019); genomic DNA isolated from faecal samples of naturally (isolation no. 43545) and experimentally (isolation no. 44331) infected chicken; genomic DNA isolated from cecum and colon of experimentally infected chicken (isolation no. 44331); hapantotypes: digital photomicrographs nos. DIC 1-13/43545, ACMV 1-11/43545, IF 1-9/43545, AP 1-12/43545, ZN IF 1-8/43545, PAS 2-3/2019 and SEM 744.75-744.79 and 745.68-745.74) and faecal smear slides with oocysts stained by ACMV staining from experimentally infected chicken (nos. 10/44331, 11/44331 and 12/44331). Specimens deposited at the Institute of Parasitology, Biology Centre of the Czech Academy of Sciences, Czech Republic.

***Site of infection***: Caecum, colon and bursa Farbricii (present study and [31]).

***Distribution***: As *Cryptosporidium* sp. ex *Struthio camelus* 2005: Brazil [36] and as *Cryptosporidium* avian genotype II: Australia [7], Brazil [56], China [57] and Vietnam [36].

***Prepatent period***: *Gallus gallus* f. *domestica*: 7 dpi; *Nymphicus hollandicus*: 8 dpi; *Anser anser* f. *domestica*: 4 dpi.

***Patent period***: At least 30 dpi in all experimentally infected birds (*Gallus gallus* f. *domestica*, *Nymphicus hollandicus* and *Anser anser* f. *domestica*)

***Representative DNA sequences***: Representative nucleotide sequences of the *SSU* (MN969957), *HSP70* (MN973934) and *actin* (MN973947) genes were submitted to the GenBank database.

***ZooBank registration***: To comply with the regulations set out in Article 8.5 of the amended 2012 version of the *International Code of Zoological Nomenclature* (ICZN) [58], details of the new species have been submitted to ZooBank. The Life Science Identifier (LSID) of the article is urn:lsid:zoobank.org:pub:593209C2-7F5B-47F9-93F3-02C81E8A747C. The LSID for the new name *Cryptosporidium ornitophilus* is urn:lsid:zoobank.org:act:FE74CF3C-6734-424B-889E-C47108DEBA60.

***Etymology***: The species name is derived from the lack of host specificity among birds and its non-infectiousness to other vertebrates.

**Description**

**Fig. 9:**
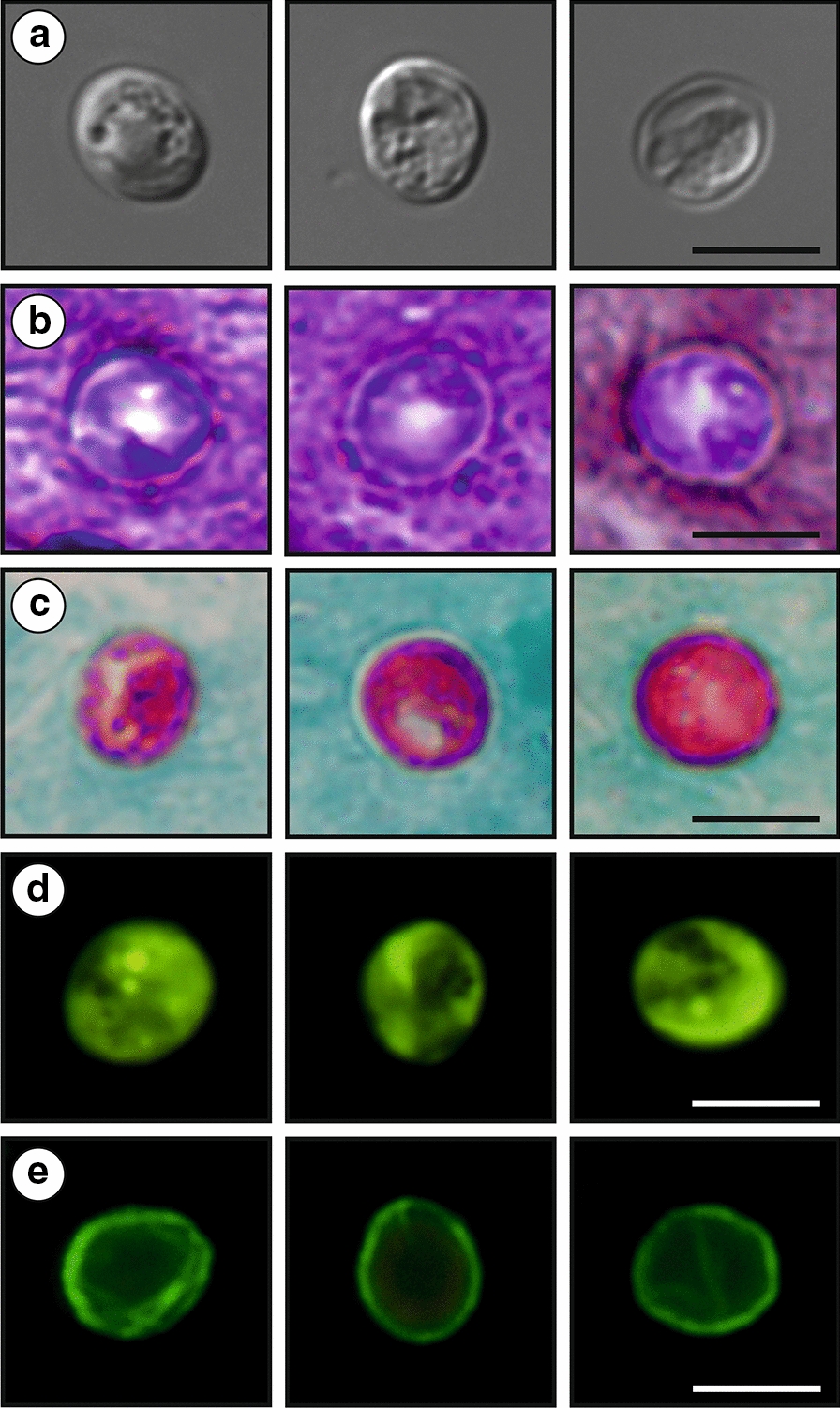
Oocysts of *Cryptosporidium ornitophilus* n. sp. visualized in various preparations. **a** Differential interference contrast microscopy. **b** Aniline-carbol-methyl violet staining. **c** Ziehl-Nielsen staining. **d** Auramine-phenol staining. **e** Labelled with anti-*Cryptosporidium* FITC-conjugated antibody. *Scale-bars*: 5 μm

Oocysts obtained from fresh feces specimens ex *Struthio camelus* ovoidal (Fig. 9), measuring 5.2–6.8 × 4.7–5.5 µm (6.1 ± 0.4 × 5.2 ± 0.2 µm) with a length/width ratio of 1.1–1.4 (1.19 ± 0.08). Oocyst wall single-layered, smooth, colorless. Micropyle and polar granule absent. Oocyst residuum present, composed of numerous small granules and one spherical globule. Four sporozoites measuring 5.5–6.6 × 0.5–0.6 µm (6.1 ± 0.3 × 0.6 ± 0.1 µm) present within each oocyst. For the measurements of other developmental stages see Table 3.

**Remarks**

Oocysts in faecal smears showed typical *Cryptosporidium* ACMV, Ziehl-Neelsen, AP staining characteristics (Fig. 9). Fixed *C. ornithophilus* n. sp. oocysts were detectable with a FITC conjugated anti-*Cryptosporidium* oocyst wall antibody developed primarily for *C. parvum* (Fig. 9). There were no statistically significant size differences between oocysts from naturally infected ostriches and oocysts obtained from experimentally infected chickens (*T*^2^ = 2.249703, *df*_1_ = 2, *df*_2_ = 97, *P* = 0.1109), geese (*T*^2^ = 0.96185, *df*_1_ = 2, *df*_2_ = 97, *P* = 0.3858) and cockatiels (*T*^2^ = 2.221246, *df*_1_ = 2, *df*_2_ = 97, *P* = 0.1139; Table 4). Oocysts of *C. ornithophilus* n. sp. are larger than those of *C. avium* (*T*^2^ = 32.522, *df*_1_ = 2, *df*_2_ = 140, *P* < 0.0001) and *C. parvum* (*T*^2^ = 147.32, *df*_1_ = 2, *df*_2_ = 78, *P* < 0.0001) and smaller than *C. proventriculi* Holubová, Zikmundová, Limpouchová, Sak, Konečný, Hlásková, Rajský, Kopacz, McEvoy & Kváč, 2019 (*T*^2^ = 161,04 *df*_1_ = 2, *df*_2_ = 90, *P* < 0.0001) and *C. galli* (*T*^2^ = 35,522, *df*_1_ = 2, *df*_2_ = 78, *P* < 0.0001). *Cryptosporidium ornithophilus* n. sp. can be differentiated genetically from other *Cryptosporidium* spp. based on sequences of *SSU*, *actin* and *HSP70* genes and on the basis of localization of life-cycle developmental stages in the host. While other bird-specific *Cryptosporidium* spp. primarily infect the proventriculus/ventriculus (*C. proventiculi* and *C. galli*) or small intestine (*C. avium*, *C. meleagridis* and *C. baileyi*) within gastrointestinal tract, *C. ornithophilus* n. sp. infects the caecum and colon.

**Table 4:** Size of *Cryptosporidium ornitophilus* n. sp. obtained from naturally infected common ostriches (*Struthio camelus*) and experimentally infected chickens (*Gallus gallus* f. *domestica*), geese (*Anas platyrhynchos* f. *domestica*) and cockatiels (*Nymphicus hollandicus*)

Host	Length (µm)	Width (µm)	Length/width ratio
	Range (Mean ± SD)	Range (Mean ± SD)	Range (Mean ± SD)
Ostrich^a^	5.24–6.77 (6.13 ± 0.35)	4.68–5.47 (5.15 ± 0.24)	1.06–1.36 (1.19 ± 0.08)
Chicken^b^	5.24–6.74 (6.13 ± 0.34)	4.71–5.48 (5.21 ± 0.23)	1.08–1.32 (1.20 ± 0.09)
Goose^b^	5.28–6.67 (6.22 ± 0.31)	4.69–5.52 (5.19 ± 0.24)	1.11–1.29 (1.18 ± 0.10)
Cockatiel^b^	5.31–6.58 (6.17 ± 0.29)	4.92–5.48 (5.19 ± 0.24)	1.09–1.28 (1.21 ± 0.12)

^a^Natural infection

^b^Experimental infection

*Note*: Length and width of 50 oocysts from each isolate were measured under DIC at 1000× magnification, and these were used to calculate the length-to-width ratio of each oocyst

## Discussion

Birds are naturally parasitized with several *Cryptosporidium* species and genotypes [16, 18]. Here, we reported the occurrence of *Cryptosporidium* spp. in ostriches farmed commercially and described *Cryptosporidium* avian genotype II as a new species. Previous studies have shown that ostriches are frequently infected with *C. baileyi* [32–34] and *C. ornithophilus* n. sp. [19, 36, 59]; however, we detected *C. ornithophilus* n. sp. and *C. ubiquitum*. The absence of *C. baileyi* could be explained by the age of the birds screened in the present study. Previous studies reported *C. baileyi* in ostriches younger than 3 months with older birds being infected rarely or not at all [32, 34]. In this study, the occurrence of *C. ornithophilus* n. sp. in birds aged 9–14 months was 4.3% (7/164), which is similar to that reported in Vietnamese ostriches older than 12 months (5.8%; [36]). The absence of *C. ornithophilus* n. sp. in birds older than three years in this study could be due to age-related resistance or immunity, as described for *C. baileyi*, *C. avium*, *C. parvum*, *C. muris* and *C. andersoni* Lindsay, Upton, Owens, Morgan, Mead & Blagburn, 2000 in various hosts [60–62], but this needs to be examined experimentally.

*Cryptosporidium ubiquitum* is not typically found in birds so our finding of five ostriches on a single farm positive for this species was unexpected. Li et al. [63] also detected *C. ubiquitum* in birds (common hill mynas, *Gracula religiosa* L.) at commercial markets in China. It is possible that the detected DNA was due to mechanical passage, not an active infection. The cohabitation of livestock, companion and wild animals can result in *Cryptosporidium* oocyst passage through non-susceptible animals without establishing infection [64–66]. We cannot exclude that some wild animals may be the source of *C. ubiquitum*. Our failure to detect oocysts also suggests that any infection was likely to be of low intensity.

Five avian *Cryptosporidium* spp. (*C. avium*, *C. baileyi*, *C. galli*, *C. meleagridis* and *C. proventriculi*) have been recognized to date, and these differ in host range, oocyst morphometry, predilection sites and course of infection. The mean size of *C. ornithophilus* n. sp. oocysts from this study (6.1 × 5.1 µm) was similar to those reported as *Cryptosporidium* avian genotype II (6.0 × 4.8 µm) by Santos et al. [31] and Meireles et al. [59], and the oocysts are morphometrically indistinguishable from those of *C. baileyi* (6.3 × 4.6 μm) [2] and *C. avium* (6.3 × 4.9 μm) [5]. Oocysts of *C. ornithophilus* n. sp. are smaller than those of *C. proventriculi* (8.4 × 6.7 µm) [6] and *C. galli* (8.3 × 6.3 μm) [4] and larger than those of *C. meleagridis* (5.0 × 4.3 μm) [3]. *Cryptosporidium ornithophilus* n. sp. infects the caecum, colon and bursa Fabricii. *Cryptosporidium baileyi* also infects the caecum, colon and bursa Fabricii (in addition to other sites in the intestine and lungs) and *C. avium* also infects the caecum (in addition to the ileum) and their oocysts are similar in size to *C. ornithophilus* n. sp. [2, 5, 31], which would make it difficult to distinguish infections without the use of molecular tools. In addition to *C. ornithophilus* n. sp., *C. baileyi* and *C. avium*, *C. meleagridis* may also develop in the colon [67, 68], but these species could be distinguished based on oocyst size. In contrast to *C. baileyi* and *C. avium*, *C. ornithophilus* n. sp. did not develop at extraintestinal sites [5, 61, 69, 70].

Similar to Ng et al. [7] and Meireles et al. [59], we found no obvious clinical symptoms or mortality in birds naturally or experimentally infected with *C. ornithophilus* n. sp. There have been reports of clinical cryptosporidiosis, including prolapse of the phallus and cloaca, enteritis and pancreatitis, in ostrich chickens, but the isolates were not genotyped [21–23, 29–31] and other species, such as *C. baileyi*, may have been the cause of disease.

Although *C. ornithophilus* n. sp. has been reported most frequently in ostriches, reports of natural and experimental infections in alexandrine, chickens, cockatiels, eclectus, galah, geese, Major Mitchell cockatoo, princess parrots, sun conure and white-eyed parakeet suggests a broad host range [7, 19, 56, 71]. The prepatent period of *C. ornithophilus* n. sp. (4–8 dpi) is similar to *C. meleagridis*, *C. baileyi* and *C. proventriculi* [6, 72–75].

Phylogenetic analyses based on *SSU*, *actin* and *HSP70* gene sequences showed that *C. ornithophilus* n. sp. is genetically distinct from known species and is most closely related to *C. baileyi* and *C. avium*. At the *SSU* locus, *C. ornithophilus* n. sp. shares 92.8% and 93.5% similarity with *C. baileyi* and *C. avium*, respectively. This is comparable to the similarity between *C. andersoni* and *C. ryanae* (91.1%) or *C. muris* and *C. suis* (93.3%). At the *actin* locus, similarities with *C. baileyi* and *C. avium* are 88.7% and 98.1%, respectively. In comparison, *C. bovis* and *C. ryanae* share 88.1% similarity and *C. parvum* and *C. erinacei* share 98.3% similarity at the *actin* locus. At the *HSP70* locus, *C. ornithophilus* n. sp. shares 91.3% and 95.6% similarity with *C. baileyi* and *C. avium*, respectively. In comparison, *C. parvum* and *C. erinacei* share 99.2% similarity at the *HSP70* locus.

*Cryptosporidium ornithophilus* n. sp. represents the 44th valid species within the genus *Cryptosporidium* (*C. alticolis* Horčičková, Čondlová, Holubová, Sak, Květoňová, Hlásková, Konečný, Sedláček, Clark, Giddings, McEvoy & Kváč, 2019, *C. andersoni*, *C. apodemi* Čondlová, Horčičková, Sak, Květoňová, Hlásková, Konečný, Stanko, McEvoy & Kváč, 2018, *C. avium*, *C. bailey*, *C. bovis* Fayer, Santín & Xiao, 2005, *C. canis* Fayer, Trout, Xiao, Morgan, Lai & Dubey, 2001, *C. cichlidis* Paperna & Vilenkin, 1996, *C. cuniculus* Robinson, Wright, Elwin, Hadfield, Katzer & Bartley 2010, *C. ditrichi* Čondlová, Horčičková, Sak, Květoňová, Hlásková, Konečný, Stanko, McEvoy & Kváč, 2018, *C. ducismarci* Traversa, 2010, *C. erinacei* Kváč, Hofmannová, Hlásková, Květoňová, Vítovec, McEvoy & Sak, 2014, *C. fayeri* Ryan, Power & Xiao, 2008, *C. felis* Iseki, 1979, *C. fragile* Jirků, Valigurová, Koudela, Křížek, Modrý & Šlapeta, 2008, *C. galli*, *C. homai* Zahedi, Durmic, Gofton, Kueh, Austen, Lawson, Callahan, Jardine & Ryan, 2017, *C. hominis* Morgan-Ryan, Fall, Ward, Hijjawi, Sulaiman, Fayer, Thompson, Olson, Lal & Xiao, 2002, *C. huwi* Ryan, Paparini, Tong, Yang, Gibson-Kueh, OʼHara, Lymbery & Xiao, 2015, *C. macropodum* Power & Ryan, 2008, *C. meleagridis*, *C. microti* Horčičková, Čondlová, Holubová, Sak, Květoňová, Hlásková, Konečný, Sedláček, Clark, Giddings, McEvoy & Kváč, 2019, *C. molnari* Alvarez-Pellitero & Sitjà-Bobadilla, 2002, *C. muris* Tyzzer, 1910, *C. nasoris* Hoover, Hoerr & Carlton, 1981, *C. occultus* Kváč, Vlnatá, Ježková, Horčičková, Konečný, Hlásková, McEvoy & Sak, 2018, *C. parvum* Tyzzer, 1912, *C. proliferans* Kváč, Havrdová, Hlásková, Daňková, Kanděra, Ježková, Vítovec, Sak, Ortega, Xiao, Modrý, Chelladurai, Prantlová & McEvoy, 2016, *C. proventriculi*, *C. reichenbachklinkei* Paperna & Vilenkin, 1996, *C. rubeyi* Li, Pereira, Larsen, Xiao, Phillips, Striby, McCowan & Atwill 2015, *C. ryanae* Fayer, Santin & Trout, 2008, *C. scophthalmi* Alvarez-Pellitero, Quiroga, Sitjà-Bobadilla, Redondo, Palenzuela, Pardós, Vázquez & Nieto, 2004, *C. scrofarum* Kváč, Kestřánová, Pinková, Květoňová, Kalinová, Wagnerová, Kotková, Vítovec, Ditrich, McEvoy, Stenger & Sak, 2013, *C. serpentis* Levine, 1980, *C. suis* Ryan, Monis, Enemark, Sulaiman, Samarasinghe, Read, Buddle, Robertson, Zhou, Thompson & Xiao, 2004, *C. testudinis* Ježková, Horčičková, Hlásková, Sak, Květoňová, Novák, Hofmannová, McEvoy & Kváč, 2016, *C. tyzzeri* Ren, Zhao, Zhang, Ning, Jian, Wang, Lv, Wang, Arrowood & Xiao, 2012, *C. ubiquitum*, *C. varanii* Pavlásek, Lávisková, Horák, Král & Král, 1995, *C. viatorum* Elwin, Hadfield, Robinson, Crouch & Chalmers, 2012, *C. wrairi* Vetterling, Jervis, Merrill & Sprinz, 1971 and *C. xiaoi* Fayer & Santín, 2009).

## Conclusions

Morphological, genetic and biological data support the establishment of *Cryptosporidium* avian genotype II as a new species, *Cryptosporidium ornithophilus* n. sp.

## Data Availability

All type material and datasets on which the conclusions of the manuscript rely, are stored in the Institute of Parasitology, Biology Centre, Czech Academy of Sciences, České Budějovice, Czech Republic. Representative nucleotide sequences generated in this study were submitted to the GenBank database under the accession numbers MN969954-MN969968 and MN973934-MN973963.

## Abbreviations

ACMV: aniline-carbol-methyl violet
AP: auramine phenol
BSA: bovine serum albumin
DIC: differential interference contrast
DNA: deoxyribonucleic acid
dpi: days post-infection
FITC: fluorescein isothiocyanate
gp60: 60-kDa glycoprotein gene
HSP70: 70-kDa heat-shock protein
ICZN: International Commission on Zoological Nomenclature
IFA: immunofluorescence assay
ML: maximum likelihood
opg: oocysts per gram
PAS: periodic acid-schiff
PCR: polymerase chain reaction
SD: standard deviation
SCID: Severe combined immunodeficiency
SSU: small subunit rRNA
ZN: Ziehl-Neelsen
